# High Mobility Group Box 1 Mediates TMAO-Induced Endothelial Dysfunction

**DOI:** 10.3390/ijms20143570

**Published:** 2019-07-22

**Authors:** Gurinder Bir Singh, Yang Zhang, Krishna M. Boini, Saisudha Koka

**Affiliations:** Department of Pharmacological and Pharmaceutical Sciences, College of Pharmacy, University of Houston, Houston, TX 77204, USA

**Keywords:** HMGB1, TMAO, tight junction, ZO2, glycyrrhizin

## Abstract

The intestinal microbe-derived metabolite trimethylamine N-oxide (TMAO) is implicated in the pathogenesis of cardiovascular diseases (CVDs). The molecular mechanisms of how TMAO induces atherosclerosis and CVDs’ progression are still unclear. In this regard, high-mobility group box protein 1 (HMGB1), an inflammatory mediator, has been reported to disrupt cell–cell junctions, resulting in vascular endothelial hyper permeability leading to endothelial dysfunction. The present study tested whether TMAO associated endothelial dysfunction results via HMGB1 activation. Biochemical and RT-PCR analysis showed that TMAO increased the HMGB1 expression in a dose-dependent manner in endothelial cells. However, prior treatment with glycyrrhizin, an HMGB1 binder, abolished the TMAO-induced HMGB1 production in endothelial cells. Furthermore, Western blot and immunofluorescent analysis showed significant decrease in the expression of cell–cell junction proteins ZO-2, Occludin, and VE-cadherin in TMAO treated endothelial cells compared with control cells. However, prior treatment with glycyrrhizin attenuated the TMAO-induced cell–cell junction proteins’ disruption. TMAO increased toll-like receptor 4 (TLR4) expression in endothelial cells. Inhibition of TLR4 expression by TLR4 siRNA protected the endothelial cells from TMAO associated tight junction protein disruption via HMGB1. In conclusion, our results demonstrate that HMGB1 is one of the important mediators of TMAO-induced endothelial dysfunction.

## 1. Introduction

Trimethylamine-N-oxide (TMAO) is a novel intestinal microbe-derived metabolite that has been recently associated with different cardiovascular pathologies [1,2,3,4,5]. Elevated levels of TMAO in plasma are shown to be associated with increased risk of major cardiovascular diseases (CVDs) in animal-model studies as well as in humans [2,6]. Elevated TMAO levels were correlated with chronic diseases associated with endothelial dysfunction and atherosclerosis as per the novel research studies [7,8,9]. Patients with chronic kidney disease have also shown increased TMAO levels in the plasma [10]. Lately, the role of TMAO in colorectal cancer, prostate cancer, and diabetes is also being reported, suggesting the important role of TMAO in the pathophysiology of diseases [11,12]. Despite clear evidence of TMAO involvement in various diseases, the underline causative mechanisms are still not clear. Modulation of platelet responsiveness, profibrotic pathways activation, and changes in cholesterol transport and excretion are a few pathways shown to be involved in TMAO associated pathogenesis [13,14,15]. Nuclear factor-kappa B (NF-kB) activation and mitogen-activated protein kinase signaling are also reported to be involved in TMAO associated pathogenesis [16]. TMAO has been reported to activate protein kinase C (PKC)/NF-kB, resulting in enhanced expression of vascular cell adhesion molecule 1 (VCAM-1) and monocyte adhesion [17]. Enhanced NF-kB expression via TMAO is also related to increased expression of inflammatory markers interleukin 6 (IL-6) and tumor necrosis factor (TNF α) [17]. Despite the progress in understanding TMAO associated pathogenesis, the exact mechanism through which TMAO causes atherosclerotic vascular diseases is currently unclear.

High-mobility group box 1 (HMGB1) is a highly conserved chromatin-binding nuclear protein with cytokine-type functions. HMGB1 is shown to be involved in several pathological developments such as inflammation, injury, infection, and a variety of diseases [18,19]. Emerging evidence indicates that HMGB1 also contributes to the pathogenesis of cardiovascular diseases. Recent reports have implicated the role of HMGB1 in endothelial dysfunction, ischemic injury, myocardial infarction, and atherosclerosis [20,21,22]. HMGB1 can activate inflammatory pathways by stimulating multiple receptors such as toll-like receptor 4 (TLR4), which is an important HMGB1 receptor [23,24,25,26]. Hence, in the present study, we tested whether TMAO upregulates HMGB1 expression in endothelial cells and contributes to endothelial dysfunction. 

## 2. Results

### 2.1. TMAO Induces Endothelial Dysfunction by Disrupting Endothelial Junction Proteins

**Figure 1:**
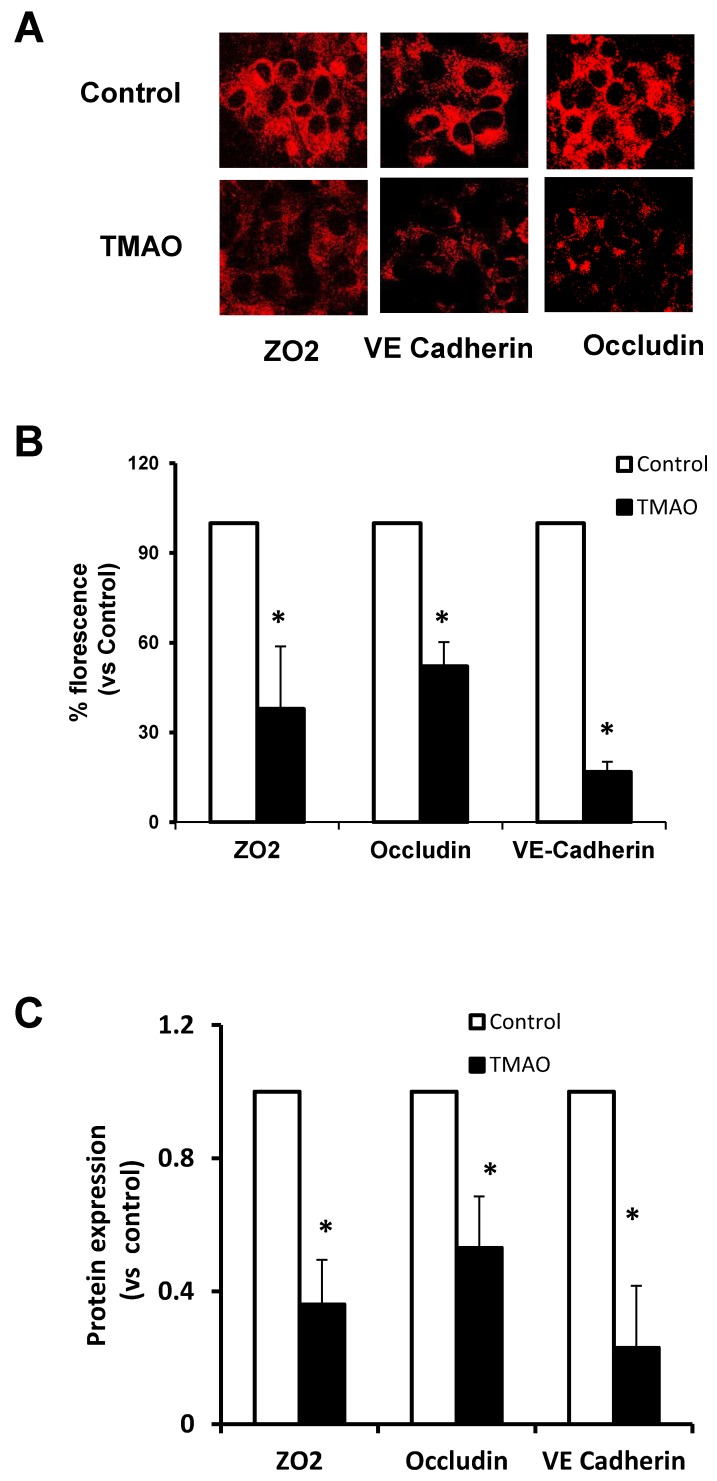
Effects of trimethylamine-N-oxide (TMAO) on tight junction proteins ZO-2, VE-Cadherin, and Occludin in EOMAs. Representative (**A**) confocal images (*n* = 5), (**B**) summarized data shows the fluorescence intensity and (**C**) Western blot (*n* = 6) show the ZO-2, VE-Cadherin, and Occludin expression in EOMAs with or without stimulation of TMAO. * significant difference from control.

The junctional architecture in endothelial cells is mainly comprised of adherent junctions and tight junctions, which are intermingled and maintain the integrity of the endothelium [27]. These junctions function as a barrier in regulating paracellular permeability and maintaining cell polarity. ZO-2, Occludin, and VE-cadherin are essential junction proteins, which are associated with junctional integrity. Downregulation of these proteins leads to junctional disruption and enhanced cellular permeability. Hence, we investigated whether TMAO-induced HMGB1 activation could lead to disassembly of junction proteins. Our immunofluorescence analysis showed that TMAO markedly decreased the expression of tight junction proteins ZO-2, Occludin, and VE-cadherin, and adhered on endothelial cell monolayers (Figure 1A,B). ZO-2, Occludin, and VE-Cadherin downregulation by TMAO was further confirmed by Western blotting (Figure 1C), suggesting that that TMAO decreased the expression of tight junction proteins. 

### 2.2. TMAO Upregulates the Expression of HMGB1, Accompanied by an Increase in Extracellular Release of HMGB1

**Figure 2:**
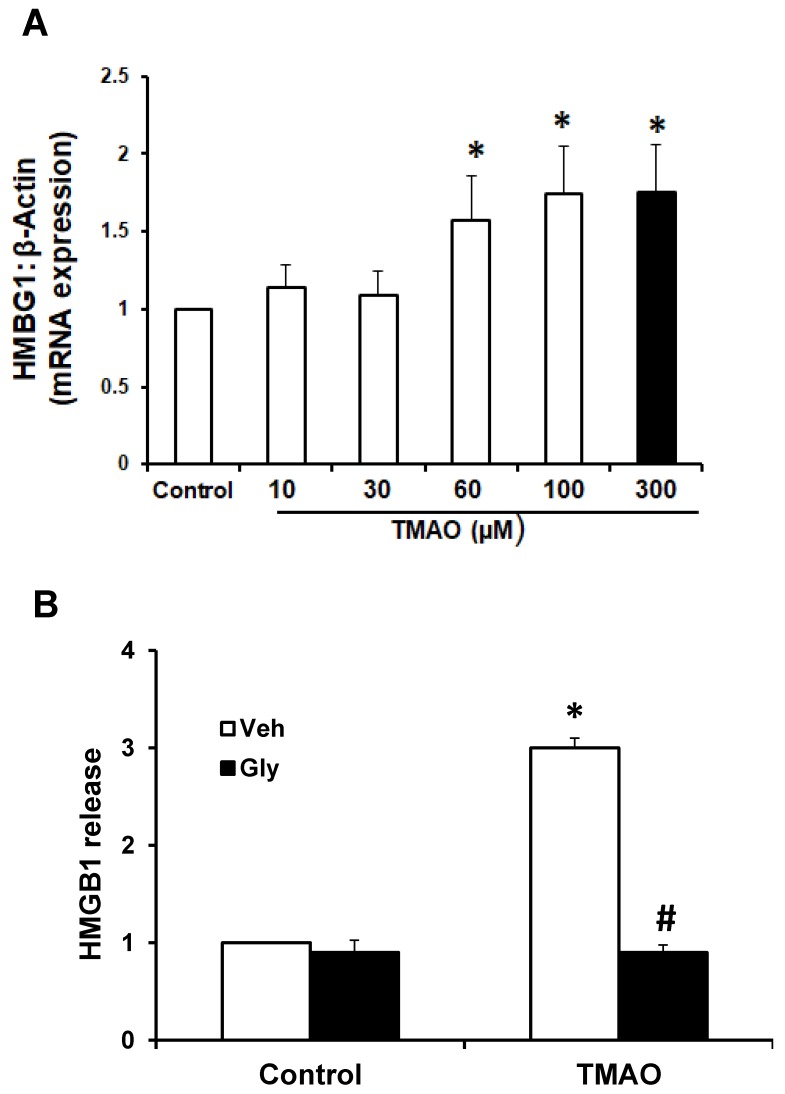
Effect of TMAO on the expression of HMGB1 in EOMAs. (**A**) RT-PCR analysis of high-mobility group box 1 (HMGB1) expression in EOMAs treated with different concentration of TMAO (*n* = 6). (**B**) Summarized ELISA data showing the HMGB1 extracellular release with or without stimulation of TMAO and/or glycyrrhizin (120 µm). Veh: vehicle, Gly: glycyrrhizin. * Significant difference from control, ^#^ significant difference from TMAO.

To examine whether TMAO can upregulate the expression of HMGB1, we detected the mRNA and protein expression of HMGB1 in EOMAs following treatment with TMAO. qRT-PCR was performed in EOMAs treated with 10, 20, 30, 60 100, and 300 μM of TMAO. We found that mRNA expression of HMGB1 significantly increased in a dose-dependent manner (Figure 2A). We selected 300 μM TMAO for treatment of EOMAs in all further experiments, as it showed a coherent increase in HMGB1 mRNA expression. Further, we tested whether HMGB1 binder glycyrrhizin attenuates the TMAO-induced HMGB1 expression. By ELISA assay, we found the TMAO treatment (300 µm) significantly increased HMGB1 release compared with control cells. Prior treatment with HMGB1 binder, glycyrrhizin significantly abolished the TMAO-induced HMGB1 release (Figure 2B). 

Next, we tested how TMAO produces HMGB1 release. Recent studies reported that activation of NLRP3 inflammasomes mediates HMGB1 release in response to a variety of exogenous and endogenous signals [28,29,30,31]. Importantly, genetic deletion or inhibition of inflammasome components severely impairs HMGB1 release during sepsis or vascular diseases [28,29,31]. Hence, we tested whether TMAO-induced NLRP3 inflammasome activation mediates HMBG1 release in EOMAs. As shown in Supplementary Figure S1, we found that TMAO increased the production of HMGB1, which was reduced by caspase-1 inhibitor, WEHD (an inflammasome component). These results suggest that TMAO-induced HMGB1 release requires NLRP3 inflammasome functionality.

### 2.3. TMAO Causes Endothelial Cell Disjunction via HMGB1

**Figure 3:**
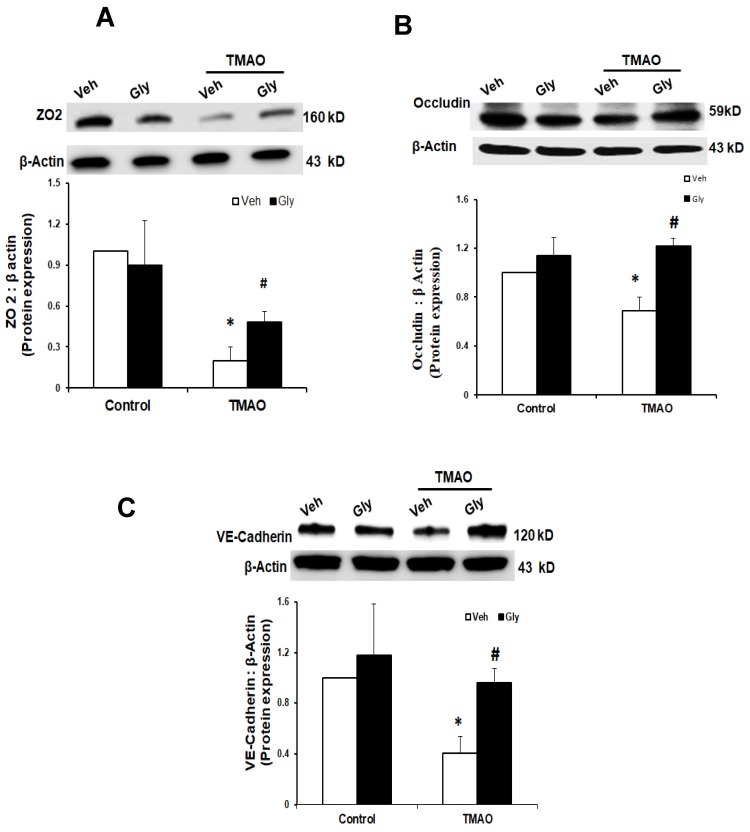
Effects of HMGB1 binder glycyrrhizin on TMAO-induced tight junction proteins ZO-2, Occludin, and VE-Cadherin in EOMAs. Representative Western blot (*n* = 3–5) images show the ZO-2 (**A**), Occludin (**B**), and VE-Cadherin (**C**) expression in EOMAs with or without stimulation of TMAO and/or HMGB1 inhibition by glycyrrhizin. Veh: vehicle. * significant difference from control, ^#^ significant difference from TMAO.

**Figure 4:**
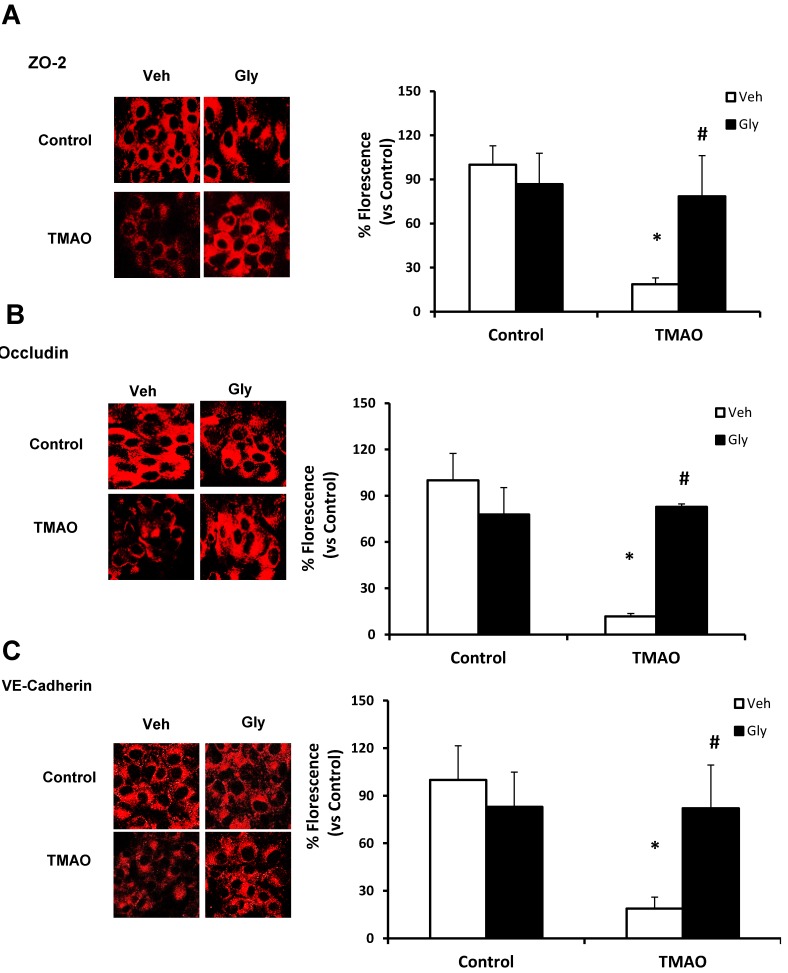
Effects of HMGB1 binder glycyrrhizin on TMAO-induced tight junction proteins ZO-2, Occludin, and VE-Cadherin in EOMAs. Representative confocal images (*n* = 3–5) show the ZO-2 (**A**), Occludin (**B**), and VE-Cadherin (**C**) expression in EOMAs with or without stimulation of TMAO and/or HMGB1 inhibition by glycyrrhizin. Veh: vehicle. * significant difference from control, ^#^ significant difference from TMAO.

Next, we examined whether HMGB1 is involved in TMAO-induced disassembly of junction proteins such as ZO2, VE-cadherin, and Occludin in EOMAs. We stimulated the EOMAs with or without stimulation of TMAO and/or glycyrrhizin. As shown in Figure 3 and Figure 4, Western blot (Figure 3) and immunofluorescence (Figure 4) analysis showed TMAO treatment significantly decreased the expression of ZO-2, Occludin and VE-cadherin compared to control cells. Prior treatment with HMGB1 binder glycyrrhizin markedly attenuated the TMAO-induced decreased expression of junctional proteins ZO-2, VE- cadherin, and Occludin in endothelial cell monolayers. In addition, we also found that treatment of EOMAs with recombinant HMGB1 significantly increased the HMGB1 expression and alters the junctional proteins (Supplementary Figure S2). These results suggest that TMAO causes junctional protein disruption via HMGB1 expression. 

**Figure 5:**
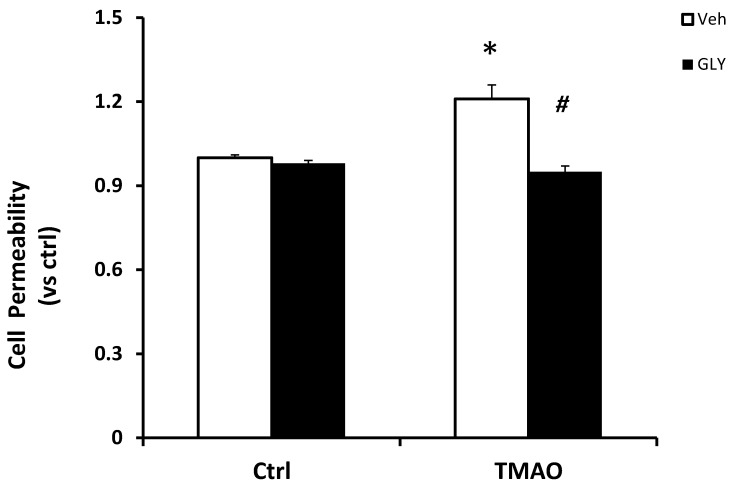
Inhibition of HMGB1 abolishes TMAO-induced cell permeability in EOMAs. Values are arithmetic means ± SEM (*n* = 6 each group) of cell permeability in EOMAs with or without stimulation of TMAO and/or glycyrrhizin. * significant difference (*p* < 0.05) compared with the values from control cells, ^#^ significant difference (*p* < 0.05) compared with the values from the TMAO-treated group.

To further determine the functional significance of TMAO associated HMGB1 activation, we examined the influence on TMAO-induced changes in the barrier function of endothelial monolayers. As shown in Figure 5, dextran flux significantly increased in EOMAs treated with TMAO compared with control cells. The presence of HMGB1 inhibitor glycyrrhizin markedly reduced TMAO-induced increase in cell permeability. Our results indicate that activation of HMGB1 by TMAO causes disruption of tight-junction proteins and alters endothelial cell permeability.

### 2.4. HMGB1 Acts in Synergy with TMAO to Upregulate the Expression of TLR4

**Figure 6:**
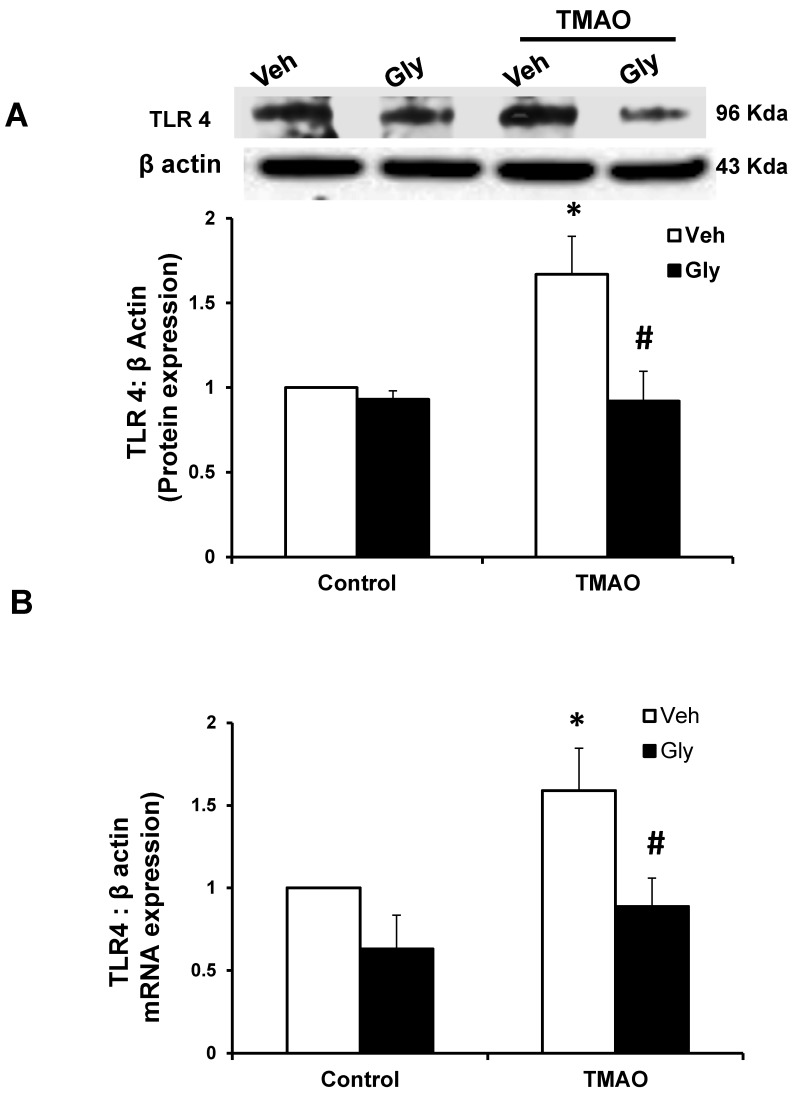
Effects of HMGB1 inhibition by glycyrrhizin on TMAO-induced toll-like receptor 4 (TLR4) expression in EOMAs. Representative Western blot images (**A**) and RT-PCR (**B**) analysis show the TLR4 expression in EOMAs with or without stimulation of TMAO and/or HMGB1 inhibitor glycyrrhizin. *n* = 5. Veh: vehicle. * significant difference from control, ^#^ significant difference from TMAO.

HMGB1 activates inflammatory pathways by stimulating multiple receptors including toll-like receptor 4 (TLR4) [23,24]. Hence, we examined whether TMAO-induced the TLR4 expression via HMGB1 in EOMAs. Our Western blot (Figure 6A) and qRT-PCR (Figure 6B) analysis showed significant increase in the expression of TLR4 in TMAO-treated cells. However, prior to treatment with glycyrrhizin, an HMGB1 binder significantly attenuates the TMAO-induced TLR4 expression (Figure 6A,B). Further, we also found that recombinant HMGB1 treatment significantly increased the TLR4 expression compared with control cells (Supplementary Figure S3). These results suggest that TMAO associated TLR4 activation is via HMGB1.

### 2.5. TMAO Causes Endothelial Dysfunction via HMGB1/TLR4 Axis

**Figure 7:**
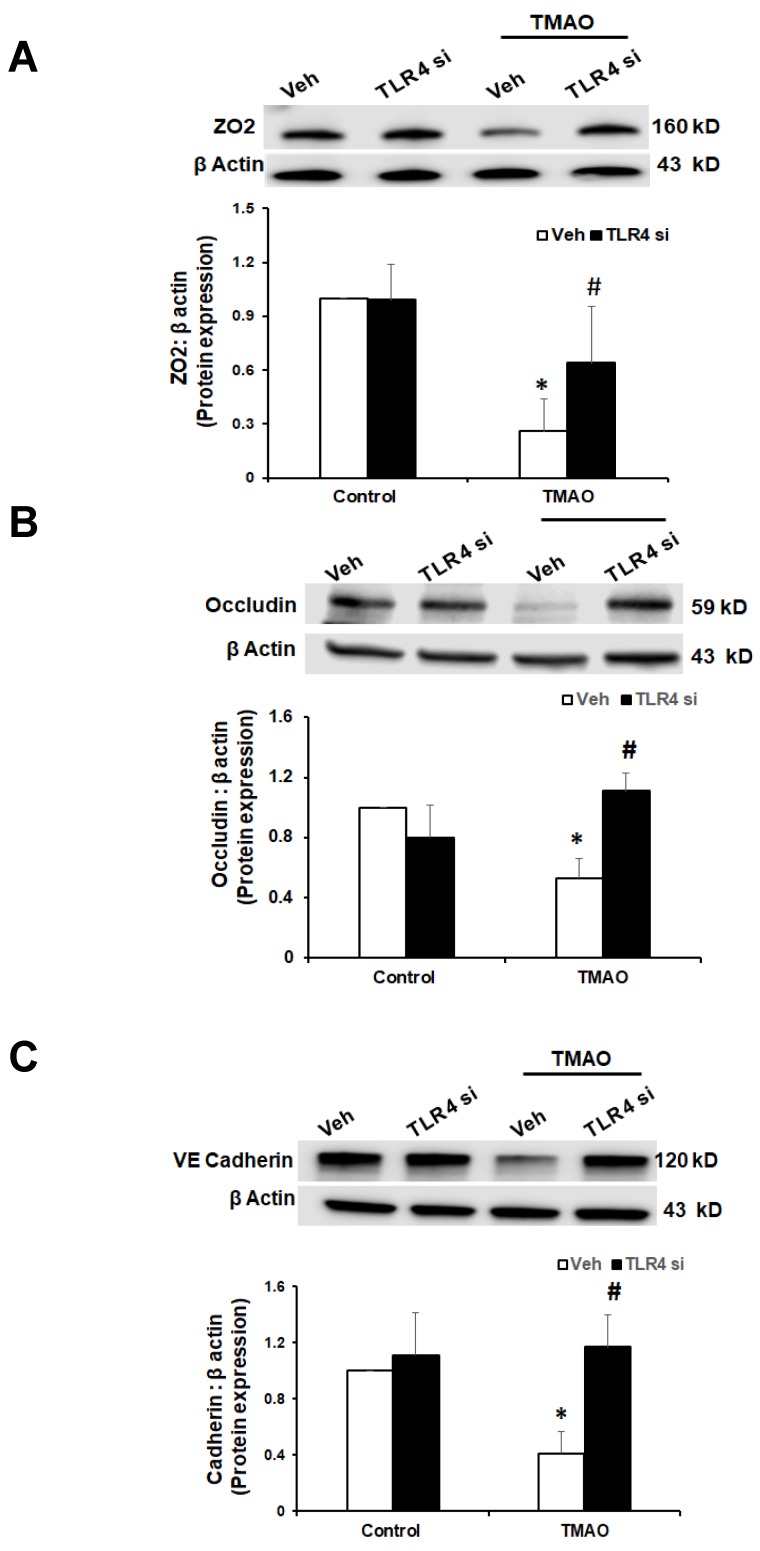
Effects of TLR4 inhibition by TLR4 siRNA on TMAO-induced tight junction proteins ZO-2, Occludin, and VE-Cadherin in EOMAs. Representative Western blot analysis shows the ZO-2 (**A**), Occludin (**B**), and VE-Cadherin (**C**) expression in EOMAs with or without stimulation of TMAO and/or HMGB1 inhibition by glycyrrhizin. *n* = 5. Veh: vehicle. * significant difference from control, ^#^ significant difference from TMAO.

**Figure 8:**
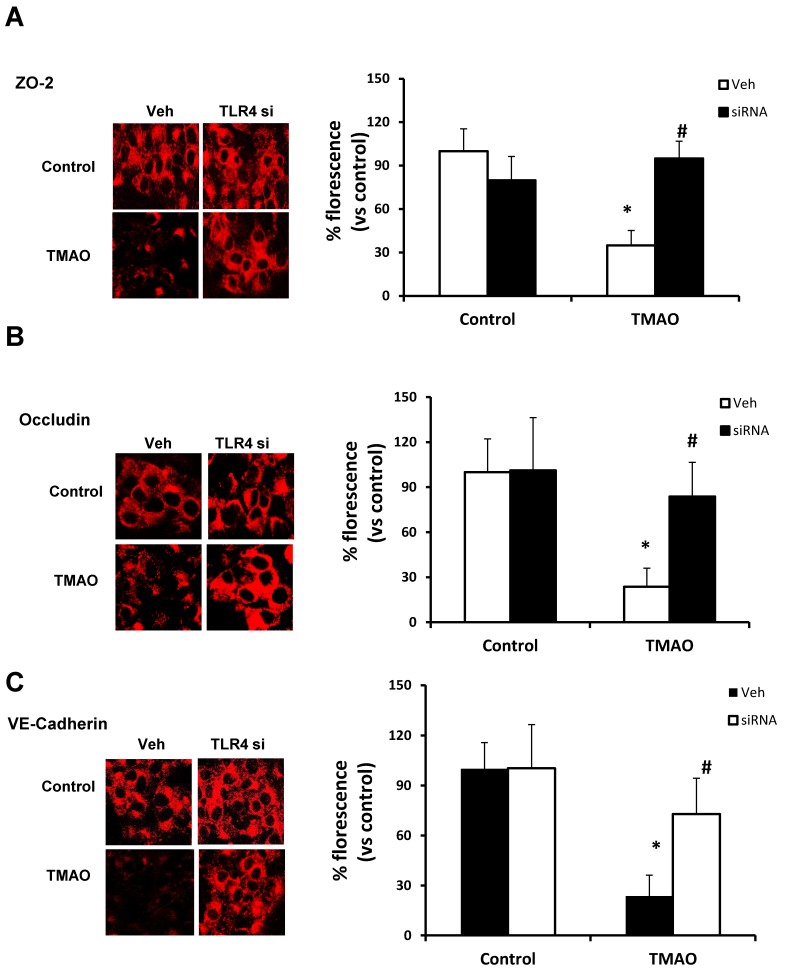
Effects of TLR4 inhibition by TLR4 siRNA on TMAO-induced tight junction proteins ZO-2, Occludin, and VE-Cadherin in EOMAs. Representative confocal analysis shows the ZO-2 (**A**), Occludin (**B**), and VE-Cadherin (**C**) expression in EOMAs with or without stimulation of TMAO and/or HMGB1 inhibition by glycyrrhizin. *n* = 5. Veh: vehicle. * significant difference from control, ^#^ significant difference from TMAO.

Our data show that TMAO upregulates HMGB1, and HMGB1 further activates TLR4. Next, we tested whether inhibition of TLR4 abolishes TMAO associated tight junction disruption on endothelial cell monolayers. We transfected EOMAs with TLR4 siRNA overnight with or without TMAO. Our Western blot (Figure 7) and immunofluorescence (Figure 8) analysis showed that silencing of TLR4 markedly improved the expression of junction proteins ZO-2, Occludin, and VE-Cadherin, confirming that TLR4 inhibition attenuates TMAO-induced junction protein disruption. 

## 3. Discussion

The present study demonstrated a novel mechanism of TMAO-induced endothelial dysfunction. We found that TMAO treatment increased the HMGB1 and TLR4 levels in endothelial cells. However, such TMAO-induced HMGB1 and TLR4 expression was abolished upon prior treatment with glycyrrhizin, an HMGB1 binder. To our knowledge, this is the first study to establish that TMAO modulated endothelial cells phenotype via activating the HMGB1 associated TLR-4 signaling pathway

Recent reports have shown TMAO to be an independent risk factor for cardiovascular disease (CVD) and established the close relationship between TMAO and CVD progression [4]. Elevated levels of TMAO are shown to promote vascular inflammation and oxidative stress, leading to atherosclerosis [32,33,34]. TMAO was also shown to promote heart failure in pressure overload-induced heart failure [34]. Endothelial dysfunction is a hallmark of vascular damage, leading to the development of atherosclerosis resulting in CVD, and is considered as an early marker for atherosclerosis. Circulating TMAO levels were shown to enhance vascular inflammation and oxidative stress and contribute to endothelial dysfunction in aged rats [2]. In addition, TMAO was shown to contribute to endothelial dysfunction via inhibiting eNOS expression and reducing NO production [9,16,32,33]. Both in in vivo and in vitro studies in vascular endothelium and smooth muscle cells, physiological levels of TMAO are shown to induce expression of cytokines and adhesion molecules, resulting in leukocyte recruitment and atherosclerosis. Previous work from our group has also explored the role of TMAO associated endothelial dysfunction via NLRP3 inflammasomes, suggesting the important role of TMAO in CVD progression [35]. However, the exact mechanisms for TMAO associated endothelial dysfunction resulting in cardiovascular diseases are still unclear. In this regard, the present study for the first time found that TMAO upregulated HMGB1 expression, and such increased HMGB1 contributes to the TMAO associated endothelial dysfunction. 

HMGB1 is non-histone DNA-binding nuclear protein with cytokine-like activity, having both intracellular as well as extracellular activities [36,37,38,39], and is shown to be involved in many pathological processes like injury, infection, inflammation, and many diseases. Recent studies have explored the role of HMGB1 in the pathophysiology of cardiovascular diseases including ischemic injury [40], myocardial infarction [41], endothelial dysfunction [32,41], and atherosclerosis [42]. HMGB1 mediated its inflammatory action by further activating various other cytokines like tumor necrosis factor-α (TNF-α), interleukin (IL)-1β, as well as IL-6 [43,44]. Apart from detection of HMGB1 in atherosclerotic plaques [45], HMGB1 levels were shown to be significantly upregulated in coronary artery disease in both diabetic as well as non-diabetic patients [46]. Above reports confirmed the role of HMGB1 in inflammation as well as in atherosclerosis. As both HMGB1 and TMAO are shown to be involved in inflammation as well as coronary artery disease, we tested whether TMAO upregulates the expression of HMGB1 in endothelial cells. In the present study, we found that TMAO increased intracellular HMGB1 expression and extracellular secretion. Recent studies have demonstrated the role of HMGB1 alone in increasing the endothelial cell monolayers permeability [47]. The endothelium forms an interface and serves as a barrier between the vessel lumen and surrounding tissue regulating tissue fluid haemostasis, angiogenesis, and vascular smooth muscle tone [27]. Adhesive properties of tight junction proteins ZO-1/2, Occludin, and VE-Cadherins control the permeability of inter-endothelial junction during vascular dysfunction [48,49,50]. Endothelial dysfunction is often characterized by enhanced endothelium permeability [51] as is found in many diseases like chronic kidney failure, venous thrombosis, stroke, heart disease, diabetes, insulin resistance, peripheral vascular disease, metastasis, tumor growth, and some viral infectious diseases [52]. This study demonstrated that TMAO decreased the expressions of tight junction proteins ZO-2, Occludin, and VE-Cadherin in cultured endothelial monolayers. Confluent endothelial cell monolayers was treated with TMAO in the presence of HMGB1 binder glycyrrhizin to further confirm the role of endothelial cell-derived HMGB1 in TMAO-induced disassembly of junction proteins. We found that glycyrrhizin decreased in the total protein expression of ZO-2, Occludin, and VE-Cadherin and prevented TMAO-induced disruption at cell junctions in the endothelial cell monolayer. 

To further study the functional significance of TMAO induced HMGB1 activation in EOMAs, we determined the role of TMAO and HMGB1 in augmentation of endothelial cell permeability. We have earlier reported that plasma proteins including albumin and visfatin contribute to increased paracellular endothelial permeability, leading to the loss of the integrity of inter-endothelial tight junctions, thus producing the impairment of renal tubular or endothelial tight junctions [53,54]. Our study suggested that TMAO treatment induced increase in permeability to dextrans in EOMAs, via activation of HMGB1, which is prevented by inhibition of HMGB1 by HMGB1 inhibitor glycyrrhizin. Our results suggest that TMAO-induced disruption of junctional proteins is dependent on HMGB1 release by endothelial cells. These results demonstrate for the first time that TMAO-induced endothelial hyper permeability is associated with HMGB1 dependent tight junction disruption. 

TLR-4 is the important HMGB1 binding receptor via which HMGB1 is shown to activate inflammatory pathways [23,24]. TLR4 activate inflammatory response by initiating innate and then adaptive immunity [55,56]. TLR4 is not only shown to recognize HMGB1, but few reports have also reported that TLR-4 further helps in release of HMGB1 suggesting important interaction between HMGB1 and TLR-4. TLR4 is well documented for mediating its role in inflammatory response. Apart from immune cells, TLR4 is also well expressed in cells of the cardiovascular system, and its role in the processes underlying inflammatory vascular diseases such as atherosclerosis, hypertension, diabetes, and other vascular inflammatory pathologies is well reported [25,26]. TLR4 expression is reported to be elevated in heart failure, hypertension, and left ventricular hypertrophy [57,58,59,60,61]. Various reports have also established the role of TLR-4 in the vascular dysfunction. TLR4 upregulation by Angiotensin II (Ang II) was shown to contribute to hypertension and vascular dysfunction through reactive oxygen species production [58]. In another report, Ang II was shown to upregulate TLR4 in hypersensitive rats further activating other pro-inflammatory genes via NF-κB signaling. Moreover, it is shown that activation of TLR4 and NF-κB increased the expression of IL-6 and adhesion molecules and decreased the activation of eNOS in atherosclerosis [62]. In a study, endothelial impairment through TLR4/NF-κB/p38 signaling was shown to play an important role in chronic heart failure [63]. Also, Qui et al., 2014 showed that TLR4 can trigger ERK1, ERK2, and NF-κB signaling pathways [64]. These reports clearly showed that HMGB1 activates TLR4 and TLR4 further activates various proinflammatory genes, contributing to inflammation and ultimately resulting in endothelial dysfunction and atherosclerosis. We attempted to determine whether HMGB1 upregulates the TLR4 pathway in TMAO treated endothelial cells. Our results confirmed that TMAO increases TLR4 expression in TMAO treated cells and HMGB1 inhibitor glycyrrhizin attenuated TMAO associated TLR4 increase. In addition, we used TLR4 siRNA to further confirm the role of TLR4 in endothelial cell permeability by measuring the expression of tight junction proteins ZO2, Occludin, and VE-cadherin, and found that TLR4 siRNA protects the endothelial cells from TMAO associated tight junction protein disruption. Therefore, our data support the view that activation of HMGB1 is a critically important signaling pathway associated with TMAO-induced endothelial barrier dysfunction injury. In conclusion, our results demonstrate that HMGB1 is one of the important mediators of TMAO-induced endothelial dysfunction. HMGB1 may be a therapeutic target for treatment or prevention of endothelial dysfunction and associated cardiovascular diseases. 

## 4. Materials and Methods

### 4.1. Cell Culture and Treatments

The mouse endothelial cell line also known as EOMA cells was purchased from ATCC (Manassas, VA, USA), which was isolated originally from mouse hemangioendothelioma. These EOMAs were cultured in Dulbecco’s modified Eagle’s medium (Gibco, Carlsbad, CA, USA) with 1% penicillin–streptomycin (Thermo Fischer Scientific, Carlsbad, CA, USA) and supplemented with 10% fetal bovine serum (Thermo Fischer Scientific), and were placed in a humidifier at 37 °C and supplied with 95% air and 5% CO_2_ mixture. Cells were trypsinized with Trypsin (Trypsin/EDTA; Sigma, St. Louis, MO, USA). The EOMAs were further diluted in Dulbecco’s modified Eagle’s medium supplemented with 10% fetal bovine serum. Cells were treated with TMAO (10, 30, 100, and 300 μm) for the TMAO stimulation, and then incubated overnight [9]. In glycyrrhizin (gly) cell groups, cells were pretreated with gly (120 µM) for thirty minutes. 

### 4.2. Immunofluorescence Analysis

Cells were grown on eight-well chamber slides. Briefly, after being treated as indicated, the cells were fixed with 4% paraformaldehyde for 15 min. Cells were then washed in phosphate-buffer saline (PBS) followed by incubation for 2 h at 4 °C with rabbit anti-ZO2 (1:200, Invitrogen), anti-Occludin (1:200, Abcam, Cambridge, CA, USA), and anti-VE-cadherin (1:200; Abcam) antibodies. The slides were incubated at room temperature with Alexa Fluor 555-labeled secondary antibody (1:500, Invitrogen) for 1 h. The slides were analyzed with sequentially scanning on an Olympus laser scanning confocal microscope (Fluoview FV1000, Olympus, Japan). Image Pro Plus software was used for the analyses of co-localization and Pearson’s correlation coefficient [35] was used to represent the co-localization coefficient.

### 4.3. Immunoblotting

Endothelial cells were washed with ice-cold PBS twice followed by homogenization in ice-cold HEPES buffer containing 25 mM Na-HEPES, 255 mM sucrose, 1 mM EDTA, and 0.1 mM phenylmethylsulfony1 fluoride (pH 7.4). After homogenization, they were centrifuged at 1000 × *g* for 10 min at 4 °C. The supernatants were snap frozen in liquid N2, and stored at −80 °C until use. Cell homogenates were denatured with reducing Laemmli SDS-sample buffer and boiled for 5 min. Homogenates were run on SDS-PAGE gel, and transferred into a PVDF membrane and blocked with 5% milk. The membranes were probed with primary antibodies anti-ZO2 (1:250, Thermo Fischer Scientific), anti-Occludin (1:500, Abcam), anti-VE-cadherin (1:500; Abcam), and TLR4 (1:500; Abcam) or β-actin. The membranes were washed with IX tris-buffered saline and 0.5% tween and incubated with secondary antibody, and then conjugated to horseradish peroxidase-labeled immunoglobulin G. The bands on the membrane were enhanced by chemiluminescence. The membranes were scanned using Licor system. 

### 4.4. qRT PCR

EOMAs were lysed, and total RNA was extracted using TRI Reagent (Sigma, St. Louis, MO, USA), according to the protocol as described by the manufacturer. A total of 1 μg of total RNA of each sample was reverse transcribed into cDNA as per the instructions of the first strand cDNA synthesis kit manufacturer (Bio-Rad, Hercules, CA, USA), followed by qRT-PCR for amplification using SYBR Green Ready Mix on a BioRad Cycler (Bio-Rad, Hercules, CA) using following primers: HMGB1 forward: CCA TTG GTG ATG TTG CAA AG; TLR4 forward: GCC CTA CCA AGT CTC AGC TA; TLR4 reverse: CTG CAG CTC TTC TAG ACC CA; β-actin forward: TGT TAC CAA CTG GGA CGA CA; β actin reverse: GGG GTG TTG AAG GTC TCA AA. Relative gene expression was calculated by the 2^−ΔΔCT^ method. 

### 4.5. Detection of Extracellular Released HMGB1

EOMAs were treated with TMAO and with or without glycyrrhizin overnight. Supernatants were collected and concentrations of released extracellular HMGB1 were measured using commercial enzyme-linked immunosorbent assay (ELISA, MyBiosource, San Diego, CA, USA) kit as per the manufacturer’s instructions. 

### 4.6. RNA interference of TLR4

Small interference RNAs (siRNAs) for toll-like receptor 4 (TLR4) were commercially obtained from Ambion, USA. The sequence for TLR4 siRNA was confirmed to be effective in silencing the TLR4 gene by the company. siRNA (20 nM) was transfected with the silentfect Lipid Reagent (Bio-Rad, Hercules, CA, USA) as per the manufacturer’s instructions. RNA interference on the expression of the targeted proteins was examined by immunoblotting using anti-TLR4 antibodies.

### 4.7. Endothelial Cell Permeability

EOMAs cells were cultured in 24-well transwell plates. Following treatment as indicated for 24 h, the transwell inserts were moved into non-used wells with 200 μL fresh media. Each insert was added with 100 μL Fluorescein isothiocyanate (FITC)–dextran (10 KDa, Sigma, St. Louis, MO, USA) and was incubated at 37 °C for 2 h. The inserts were then removed and fluorescent intensity was determined at excitation/emission of 485/530 nm using a fluorescent microplate reader (FL × 800, BIO-TEK Instruments). The arbitrary fluorescence intensity was used to calculate the relative permeability [35].

### 4.8. Statistics

All data obtained from at least three independent experiments are presented as means ± SEM. Significant differences between and within multiple groups were examined using analysis of variance (ANOVA) for repeated measures, followed by Duncan’s multiple-range test. *p* < 0.05 was considered statistically significant.

## Supplementary Materials

Supplementary materials can be found at https://www.mdpi.com/1422-0067/20/14/3570/s1.
